# Emerging Technologies for Future Sensor Networks—Selected Papers from ICGHIT 2019

**DOI:** 10.3390/s19183854

**Published:** 2019-09-06

**Authors:** Byung-Seo Kim, Sung Won Kim, Chi Zhang, Yuanxiong Guo, Tariq Umer

**Affiliations:** 1 Department of Software and Communications Engineering, Hongik University, Sejong 30016, Korea; 2 Department of Information and Communication Engineering Yeungnam University, Gyeongsan 38541, Korea; 3 School of Information Science and Technology, University of Science and Technology of China, Hefei 230026, Anhui, China; 4 Department of Information Systems and Cyber Security, University of Texas at San Antonio, San Antonio, TX 78249, USA; 5 Department of Computer Science, COMSATS Institute of Information Technology, Wah Cantt 47040, Pakistan

## 1. Introduction

The International Conference on Green and Human Information Technology (ICGHIT) is an international conference focusing on green and information technologies oriented toward humanity. The goal of ICGHIT is to form a platform to seek the advancement of green technology and human related IT in an interdisciplinary manner. The main topics of the conference are green Information Technology (IT), communications, the Internet of Things (IoT), computer and network security, multimedia and signal processing, control and intelligent systems, and green IC system design. Many papers on advanced technology will be presented and various products from multiple companies will be exhibited. The conference hopes to provide good chances for discussion about the topics for technical exchange and advancement, cooperation among the participants, and local development of the country in which the conference is held. We believe that green technology and human IT can be synergized to enhance human welfare and humanity in the present and in future technological societies. The philosophy of the conference is the achievement of the dream of IT, which is to improve human welfare and happiness. Therefore, ICGHIT emphasizes humanity as well as technology. 

The 7th ICGHIT was held in Kuala Lumpur, Malaysia on January 16th–18th 2019. This year’s conference theme is “Challenges and Opportunities in the Post-AI Era”. The latest technologies from Artificial Intelligence (AI) already pervade our daily life regardless of our recognition. They give big challenges and great opportunities at the same time. Centering on this theme, we provide an exciting AI program: Hands-on experience-based tutorial sessions, and special sessions covering research issues & directions with applications from both theoretical and practical viewpoints. Furthermore, the “Future Sensor Networks” topic was added as a research topic in the Call-for-Paper. Detailed research items of this topic include Architecture and Protocols, Sustainable Sensor Networks, Information centric Sensor Networks, Blockchain-based Secure Sensor Networks, and AI-based Self-evolving Sensor networks. All papers published in this Special Issue (SI) are selected from the papers submitted for “Future Sensor Networks” topic. The editorial board of ICGHIT 2019 selected a total of 14 high quality papers among 218 submissions. The selected papers were required to extend 50% more from the conference paper, and throughout extensive peer review process of Sensors journal including 2nd and even 3rd revisions, only six papers are finally published for this SI. In the next section of this editorial, the six published papers are briefly introduced.

## 2. Contributions 

Chirp Spread Spectrum (CSS) technology is one of the communication technologies for a long-range communication with high data rate in the Internet-of-Things (IoT) system. In addition, the two-path successive relaying (TPSR) protocol has been proposed in order to increase spectral efficiency and to extend IoT devices’ transmission coverages. The CSS-based TPSR protocol for IoT system is proposed in a paper, titled: “Inter-Relay Interference Mitigation for Chirp-Based Two-Path Successive Relaying Protocol” [1]. In the paper, the cross-correlation coefficient (CCC) has been derived mathematically according to a separating bandwidth in a given total bandwidth. Then, one separating bandwidth that guarantees the transmission performance is allocated to the primary relay by considering a single relay CCC (SR-CCC). Another separating bandwidth that guarantees the orthogonality from the primary relay is allocated to the secondary relay by considering the inter-relay CCC (IR-CCC). Since the IR-CCC means a degree of similarity between these two relays, it is possible to mitigate the inter-relay interference (IRI) effect within the same bandwidth by allocating orthogonal separating bandwidths to each relay. Simulation results show that the proposed scheme can improve the transmission performance by mitigating the IRI effect even in high IRI environments.

For resolving the issues like energy efficiency and stability of IoT Mobile systems, the enhancement of 3-dimensional group management medium access control (3-D GM-MAC) is proposed in the paper, titled “Enhanced 3-D GM-MAC Protocol for Guaranteeing Stability and Energy Efficiency of IoT Mobile Sensor Networks” [2]. The 3-D GM-MAC protocol has been designed for cases where both the sensor nodes and sinks are mobile. The sensor nodes are assigned with group numbers based on the distance to the sink, and they have a hierarchical structure with a tree shape. A sensor node sends data to the sensor node of the next upper group. The sensor node of the next upper group has a group number that is smaller by one than its group number. The paper proposes three methods to enhance the current 3-D GM MAC protocol. First, a new buffer threshold equation was derived. Second, a fixed node is assigned to whole wireless sensor networks (WSNs). The third method of improving the stability of 3-D GM-MAC is to adopt an advanced method for the management of the group number. Based on the extensive simulation works, it is shown that the energy efficiency of the proposed method increased by approximately 23.4%, when compared with the original method.

Information centric networking (ICN) is one of the candidates for future Internet technologies. In ICN, instead of using IP address as a destination, the names of the required contents are used. For example, if a node needs certain content or information such as movie, music, and sensed data, it sends an Interest packet throughout the network and if any node has the content, it will forward the content to the node. Therefore, in such environments with dynamic topology changes and massive devices such as an IoT environment, ICN may enhance the performance of these networks. There are two papers presented in this special Issue: “Hierarchical Name-Based Mechanism for Push-Data Broadcast Control in Information-Centric Multihop Wireless Networks” [3], and “NINQ: Name-Integrated Query Framework for Named-Data Networking of Things” [4]. In [3], an ICN protocol for two application scenarios such as smart buildings and vehicular networks is proposed. The protocol includes a robust content namespace, device namespace, and amendments to the data packet format and unsolicited data policy of the forwarding engine of conventional Named Data Networking (NDN) platforms. The proposed method controls the number of unnecessary data packets in the network which are proved by extensive simulations using an ndnSIM simulator. In [4], the paper proposes a flexible, expressive, and secure query mechanism that supports content retrieval as well as control and configuration command exchange among various nodes in a smart building. Through the Comparative studies, the proposed mechanism outperforms the most recent naming schemes in terms of Interest Satisfaction Rate (ISR), Command Satisfaction Rate (CSR), number of packets processed in the network, energy consumption, and average delay.

As one of the IoT applications, Indoor Positioning is studied by a paper, titled “IPSCL: An Accurate Indoor Positioning Algorithm Using Sensors and Crowdsourced Landmarks” [5]. In the paper, to increase the accuracy of the indoor positioning, smartphone built-in sensors and Bluetooth beacon-based landmarks are utilized. While the proposed method uses the gyroscope and the magnetometer sensors of the smartphone, the readings of these sensors are occasionally different from each other and as a result, the heading direction of moving people as calculated using the sensors is inconsistent. To reduce the differences, a reference point is required and for this, the paper exploits the Bluetooth beacon-based landmark approach which is a datum point that is visible to serve as a reference by being differentiated from other points and exhibiting consistent patterns. Through extensive experimental evaluations, it is shown that the proposed technique facilitates the acquisition of accurate heading direction and coordinates of the user.

Since security is considered as one of the fatal points of IoT and recently Block Chain has drawn a lot of attention, research efforts to combine Block Chain into IoT systems have been made. A paper in this Special Issue, titled “Packet Key-Based End-to-End Security Management on a Blockchain Control Plane” [6], proposes such a method to use Block Chain for IoT systems. It proposes a packet key-based security management scheme over the blockchain control plane to improve the current session key-based security system, so that it can overcome the limitation that the existing vertical model confronts in solving end-to-end security management. The proposed management structure is close to the administrative control based on what the Software-Define-Networking (SDN)-based horizontal model operates with. However, unlike conventional SDN, the proposed scheme utilizes one of the most innovative features of the blockchain, that there is no central server running.
